# Extracellular vesicles, the emerging mirrors of brain physiopathology

**DOI:** 10.7150/ijbs.79063

**Published:** 2023-01-01

**Authors:** Amanda Cano, Miren Ettcheto, Mireia Bernuz, Raquel Puerta, Ester Esteban de Antonio, Elena Sánchez-López, Eliana B. Souto, Antonio Camins, Mercè Martí, María Isabel Pividori, Mercè Boada, Agustín Ruiz

**Affiliations:** 1 Ace Alzheimer Center Barcelona - International University of Catalunya (UIC), Barcelona, Spain; 2 Biomedical Research Networking Centre in Neurodegenerative Diseases (CIBERNED), Madrid, Spain.; 3 Institute of Nanoscience and Nanotechnology (IN2UB), Barcelona, Spain.; 4 Department of Pharmacy, Pharmaceutical Technology and Physical Chemistry, Faculty of Pharmacy and Food Sciences, University of Barcelona, Spain.; 5 Department of Pharmacology, Toxicology and Therapeutic Chemistry, Faculty of Pharmacy and Food Sciences, University of Barcelona, Spain.; 6 Biosensing and Bioanalysis Group, Institut de Biotecnologia i de Biomedicina (IBB-UAB), Mòdul B Parc de Recerca UAB, Campus Universitat Autònoma de Barcelona, 08193 Bellaterra, Spain.; 7 Grup de Sensors i Biosensors, Departament de Química, Universitat Autònoma de Barcelona, 08193 Bellaterra, Spain.; 8 Unit of Synthesis and Biomedical Applications of Peptides, IQAC-CSIC, 08034 Barcelona, Spain.; 9 Department of Pharmaceutical Technology, Faculty of Pharmacy, University of Porto, Porto, Portugal.; 10 REQUIMTE/UCIBIO, Faculty of Pharmacy, University of Porto, Porto, Portugal.

**Keywords:** Exosomes, extracellular vesicles, Alzheimer's disease, Parkinson's disease, glioblastoma, multiple sclerosis, amyotrophic lateral sclerosis, neurodegenerative diseases

## Abstract

Extracellular vesicles are secreted by a wide variety of cells, and their primary functions include intercellular communication, immune responses, human reproduction, and synaptic plasticity. Their molecular cargo reflects the physiological processes that their cells of origin are undergoing. Thus, many studies have suggested that extracellular vesicles could be a promising biomarker tool for many diseases, mainly due to their biological relevance and easy accessibility to a broad range of body fluids. Moreover, since their biological composition leads them to cross the blood-brain barrier bidirectionally, growing evidence points to extracellular vesicles as emerging mirrors of brain diseases processes. In this regard, this review explores the biogenesis and biological functions of extracellular vesicles, their role in different physiological and pathological processes, their potential in clinical practice, and the recent outstanding studies about the role of exosomes in major human brain diseases, such as Alzheimer's disease (AD), Parkinson's disease (PD), multiple sclerosis (MS), amyotrophic lateral sclerosis (ALS), or brain tumors.

## 1. Introduction

Intercellular communication involves various molecules such as RNA, amino acids, lipids, metabolites, and proteins, which are commonly packaged into extracellular vesicles (EVs) 1. EVs are membrane-enclosed structures surrounded by a phospholipid bilayer membrane secreted by different cells, whose constituents reflect their cells of origin and their physiological and pathological processes 1. Several reports have highlighted that these EVs acts like messengers in intercellular communication, conveying information to neighboring or remote cells.

EVs encompass three major subcategories: apoptotic bodies, microvesicles, and exosomes. The largest of the EVs are the apoptotic bodies and microvesicles. These EVs directly originate from the invagination of the plasma membrane in an endocytic process. Exosomes, the smallest EVs, are located in multivesicular bodies (MVBs) formed intracellularly by multiple invaginations of the late endocytic membrane 2.

A growing number of studies have suggested that EVs could be a promising tool as new biomarkers for many diseases, mainly due to their biological relevance and easy accessibility to a broad range of body fluids 3. Moreover, it has been reported that cells suffering from any pathological process, such as cancer cells, release more EVs than healthy cells 4,5. Thus, the appearance of EVs with specific cargoes and surface markers of a wide variety of pathological processes has been described in different human diseases such as AD 6, cancers 7, cardiovascular diseases 8, sarcoidosis 9, or prion diseases 10, among others.

In this review, we explore the biological functions of EVs, their role in different physiological and pathological processes, their potential in clinical practice, and the recent findings about the role of EVs in major human brain diseases.

## 2. Biogenesis and main hallmarks of EVs

**Figure 1:**
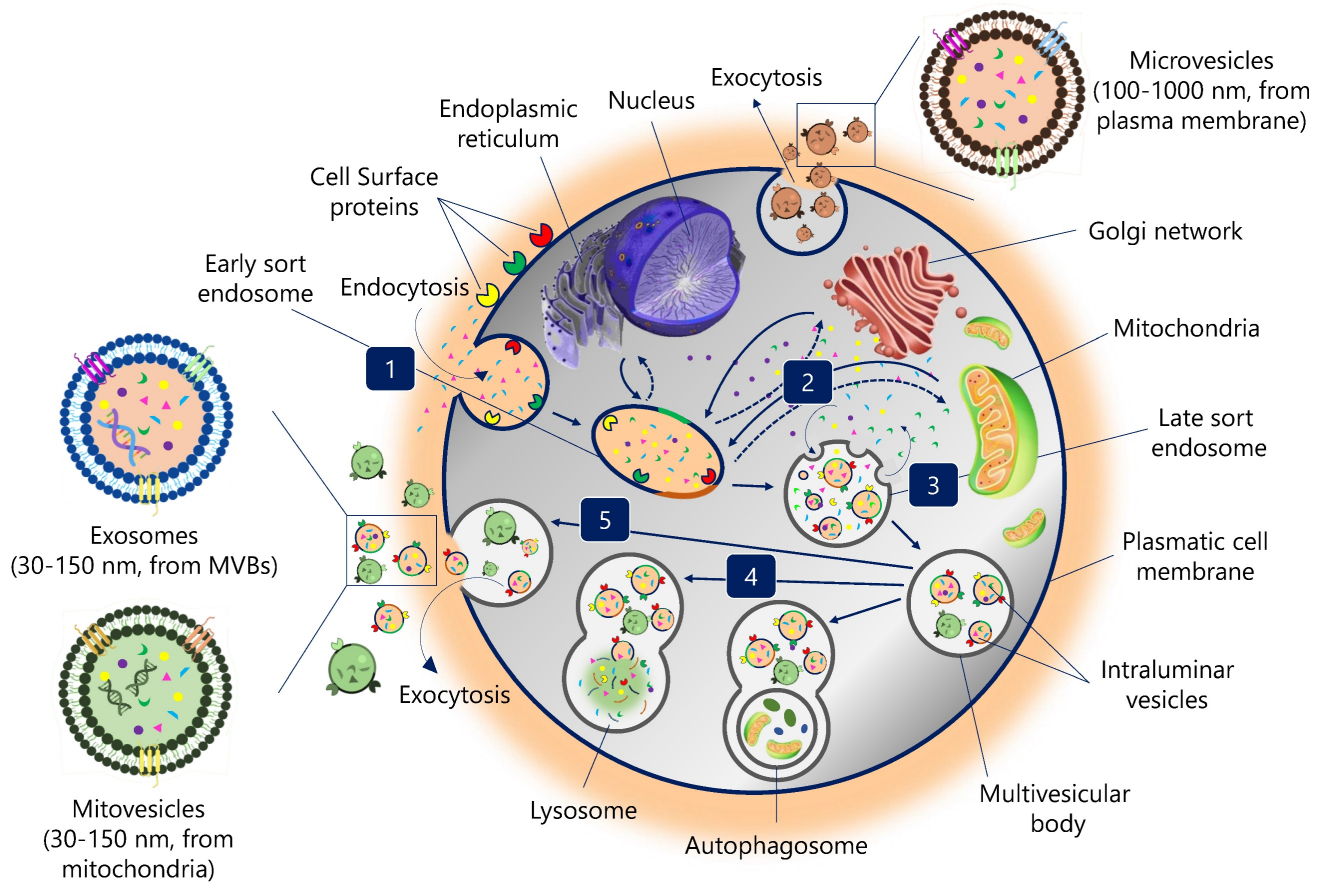
** Biogenesis pathway of extracellular vesicles. 1 Endocytosis.** Invagination of plasma membrane, involving extracellular constituents together with cell surface proteins. The resulting endosome membrane presents with outside-in plasma membrane orientation. **2 Formation of the endosome.** This budding process leads to the formation of early sort endosomes (ESEs) or possible fusion of the bud with endosomes pre-formed by the endoplasmic reticulum (ER), Golgi network (GN) and mitochondria. Then, ESEs give rise to late sort endosome (LSEs). **3 Formation of the multivesicular body**. Second invagination in the LSE leads to the generation of intraluminar vesicles (ILVs), which in turn give rise to multivesicular bodies (MVBs) with defined cargo of ILVs (future exosomes). **4 MVBs fusion.** The MVB can fuse either with lysosomes or autophagosomes to be degraded. **5 Exocytosis.** MVBs can be transported to the plasma membrane through the cytoskeletal and microtubule network and dock on the luminal side of the plasma membrane. Exocytosis involves the release of the extracellular vesicles, like exosomes, microvesicles and mitovesicles (extracellular vesicles of mitochondrial origin) with a similar lipid bilayer orientation as the plasma membrane.

EVs biogenesis is initiated by plasma membrane invagination of the origin cell. The first invagination forms a structure that includes cell-surface proteins and soluble molecules associated with the extracellular matrix, such as ions, metabolites, peptides, or lipids (**Figure 1**) 11. This leads to the formation of a new early endosome (ESE). The primary endosome may also fuse with ESEs pre-formed by the endoplasmic reticulum (ER), Golgi network (GN), and mitochondria. Likewise, the ESEs could also fuse with the ER and TGN, which could explain how the endocytic cargo reaches these organelles and *vice versa*. Then, ESEs evolve into late endosomes (LSEs), where the exchange between the components of the endosome and those present in the cytosol is significantly increased. The second invagination of LSEs leads to the generation of intraluminal vesicles (ILVs). In this step, proteins (originally belonging to the cell surface) could be distinctly distributed among ILVs. Moreover, depending on the invagination volume, ILVs of different sizes and variable content could be found 11.

Encapsulating several ILVs (future EVs) generates MVBs, also known as multivesicular endosomes 2. Then, MVBs can follow several routes: (i) MVBs can fuse with autophagosomes, whose content will undergo degradation in the lysosomes; (ii) MVBs can directly fuse with lysosomes for degradation, and the cells could recycle the degradation products; (iii) MVBs that escape this trajectory are transported to the plasma membrane through the microtubule network and the cytoskeleton 2. On the luminal side of the plasma membrane, MVB-docking proteins facilitate a docking process between the MVB and the lipid bilayer. Finally, exocytosis results in the release of EVs (**Figure 1**).

EVs can be divided into different types based on their size and origin: exosomes are 30-150 nm in size and derived from intracellular vesicles (multivesicular bodies, a form of late endosome); microvesicles directly bud off the plasma membrane and are usually between 100 and 1000 nm; mitovesicles, EVs with mitochondrial content and similar size and route than exosomes; while apoptotic bodies are generated from dying cells and are generally between 100 and 5000 nm 12.

EVs frequently contain apoptosis-linked gene 2 interacting protein X (ALIX), endosomal sorting complexes required for transport proteins (ESCRT), Ras-related protein GTPase Rab, soluble N-ethylmaleimide-sensitive factor attachment protein receptor (SNARE), tumor susceptibility gene 101 (TSG101), syntenin-1, syndecan-1, ceramides, phospholipids, sphingomyelinases, and tetraspanins 13. In addition, CD9, CD81, CD63 or flotillin are commonly used as surface biomarkers for EV tracking. However, it is remarkable that these molecules are not only present in exosomes surface and they are also found, for example, in multinucleated giant cells 14. Likewise, EV cargo can contain DNA, RNA, metabolites, amino acids, different types of cell surface proteins, and intracellular proteins 15.

The heterogeneity of EVs is large and is mainly determined by four factors 2,16: (i) EVs' size, which could be due to uneven invagination of the MVB's limiting membrane, affecting the different amounts of EVs content; (ii) e EVs' content that may involve extracellular matrix, membrane, cytosolic and nuclear proteins, lipids, metabolites, and nucleic acids (noncoding RNA species and DNA). The inherent biology conditions this cargo and the microenvironment of the cells of origin; (iii) EVs' origin: Different cells and organs use EVs as a tool for intercellular communication. Heterogeneity can be found in the tissue of origin, giving them different properties such as uptake by specific cell types or tropism toward certain organs; (iv) functional impact: The effects of EVs on target cells can be different due to the diversity of cell surface receptor expression. This can result in EVs inducing cell survival, apoptosis, or immunomodulatory functions 17.

## 3. Physiological functions of EVs

The biological functions of EVs are complex and diverse. Some of their cellular pathways are not yet completely understood, as well as their paracrine, autocrine, or endocrine activity 18,19. However, it is well known that EVs play a vital role in cellular intercommunication 20. In this process, both EV uptake and secretion pathways coexist, resulting in a mixed production of EVs (newly synthesized or recycled) over time 2,20. This horizontal transfer of material mediated by EVs exchange causes various phenotypic and molecular alterations in the receptor cells of the different organs and tissues. Likewise, it has also been reported that the “turnover rate” of internalized EVs may vary depending on the metabolic state of the recipient cell 2. Thanks to the wide range of molecules that EVs can carry, this cellular communication strategy leads to much more complex events than the signaling mediated by a single ligand and receptor. Several mechanisms have been described by which bioactive molecules transported by EVs affect target cells: (i) epigenetic reprogramming of recipient cells through delivery of functional proteins, lipids, ions, and RNAs; (ii) transfer of activated receptors to recipient cells; and (iii) direct stimulation of target cells through surface-bound ligands 11.

**Figure 2:**
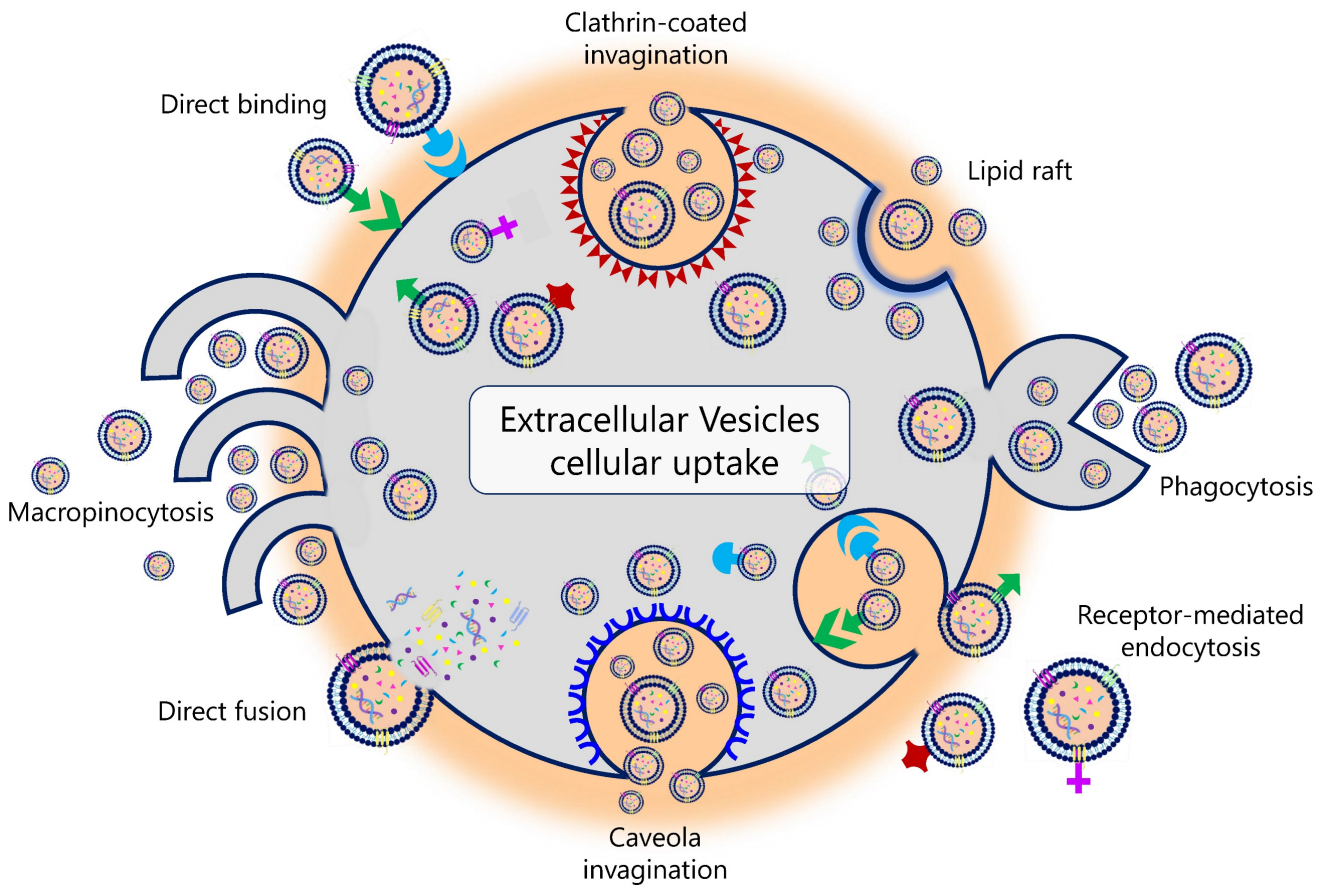
Physiological pathways of extracellular vesicles' biological uptake.

Different mechanisms are associated with EV uptake (**Figure 2**). For example, it has been described that the uptake of EVs in human pancreatic cancer cells is caused by macropinocytosis through oncogenic signals induced by the expression of mutant KRAS 21,22. In human melanoma cells, this uptake is carried out through the fusion of the EV with the plasma membrane 23. It is currently unknown why this variation exists and whether the uptake of EVs results in a different functionality from that of the EV constituents themselves 24. Tracing the intercellular exchange of EVs is also a great challenge today. Several* in vivo* studies explored different genetic strategies in animal models. Under physiological conditions, these studies demonstrated that EVs do not usually deliver mRNA to a recipient cell. In contrast, this delivery, promoted by the activation and expansion of EV-producing immune cells, was increased in mouse models of acute and chronic inflammation 25. Likewise, therapeutic interventions can also influence EV uptake. In this regard, Parolini *et al*. showed that the inhibition of proton pumps led to an inhibition of EV uptake. The acidification of the cellular pH increased the EV release and uptake in melanoma cell lines 26.

EVs have also been shown to be involved in maintaining and improving synaptic plasticity and neurotransmission in the adult brain, which is crucial for cognitive function 27. It has been shown that EVs modulate synaptic activity in rapid and progressive manners and have some similarities with synaptic vesicles in biogenesis, content, and function Together with neurons, glial cells, such as astrocytes and microglia, are mediated by neural synaptic plasticity processes 28. Active zones on the presynaptic plasma membrane are responsible for depositing synaptic vesicles into the cytoplasm of the nerve terminal. This synaptic vesicle exocytosis is typical of synaptic neurotransmission 27. However, signal transduction of neurons through the secretion of EVs can also induce a wide range of neurobiological functions, including synaptic plasticity 29. In this regard, Bahrini *et al.* demonstrated that PC12 cell-derived EVs could stimulate synaptic pruning by enhancing the complement component 3 level in microglial MG6 cells. Furthermore, it has also been described that neuronal-derived EVs might affect synaptic plasticity by managing the number of AMPA receptors for glutamate transmission 30. Additionally, increased secretion of EVs derived from cortical neurons, which carry neurotransmitter receptors, led to enhanced glutamatergic activity 30.

Numerous mechanisms of intercellular communication are orchestrated by neuron-glia homeostasis balance. EVs are involved in the physiological interactions between all the cells that compose the neurovascular unit. In addition, EVs are present in neuronal tissue's intrinsic development and protection processes 27. Evidence has indicated that communication between glia and neurons through EVs is required for neuronal growth and cell survival 31,32. The protection of neurons by astrocyte-derived EVs is dependent on astrocyte-derived EV PrP transport into neurons, a physiologically important receptor protein that protects against oxidative stress in the CNS 33. Other studies indicate that oligodendrocyte- and microglia-derived EVs transfer several enzymes to neurons, thus contributing to different energy metabolism pathways 34,35. All these processes comprise normal brain function.

The role of EVs in innate immune responses and their homeostasis has been extensively studied 36-41. Several mechanisms have been described by which EVs contribute to this physiological function: (i) induction of signaling pathways by ligands present on the surface of EVs; (ii) transfer and presentation of antigenic peptides; (iii) gene-expression promoted by EV miRNA cargo; and (iv) the delivery of DNA-inducing cyclic GMP-AMP synthase stimulators of interferon genes in recipient cells, in which sensing of cytosolic DNA triggers the expression of a type I IFN response and inflammatory gene expression 2. EVs secreted by antigen-presenting cells (APCs) carry p-MHC-II and costimulatory signals. This directly presents the peptide antigen to specific T cells to induce their activation. However, T-cell stimulation by APCs is more effective than EV stimulation 42-44. The role of EVs in antigen presentation has also been described in the context of bacterial infection 2. EVs could enhance antibacterial immune responses by promoting bacterial antigen presentation from macrophage-derived EVs, which subsequently influences the adaptive immune response 45. Likewise, Patil *et al.* also demonstrated the opsonization role of EVs in mediated phagocytosis of dead cells in the injured heart 46. The nucleic acids' EV cargo, mainly DNA and miRNAs, has also been identified to regulate innate and adaptive immune responses. EVs DNA has been shown to play a role in cancer progression, and miRNA transfection may also regulate the immune response by influencing gene expression and signaling pathways in recipient cells 2.

EVs are present in breast milk, amniotic fluid, and semen, suggesting that EVs possess putative functions in human reproduction, pregnancy, and embryonic development 2. Seminal plasma EVs have been implicated in sperm maturation 47. The miRNA signature of EVs has been found to play a vital role in the expression of interleukins involved in genitalia-resident immunity and the prevention of infections in the placenta 48. Moreover, miRNAs and the protein cargo of plasma EVs dynamically evolve during pregnancy 49. EV-derived miRNAs in breast milk also seem to promote postnatal health and growth 50.

Finally, another biological function of natural EVs is wound healing. EVs derived from bone marrow mesenchymal stem cells have been shown to activate several signaling pathways in the wound healing process, such as STAT3, ERK, and Akt 51. Likewise, they are also implicated in the expression of many wound healing-related growth factors, such as nerve growth factor, insulin-like growth factor 1, stromal cell-derived factor 1, or hepatocyte growth factor 51. In addition, several studies have pointed out the crucial role of EVs in controlling the inflammation associated with wound healing. The release of EVs by mesenchymal stem cells creates a suitable microenvironment through the transfer of EV miRNA. This micro RNA could regulate the expression of important immuno-modulators that fine-tune, for example, the TLR-induced NFκB signaling pathways and their downstream targets, thus reducing the inflammatory process surrounding wounds without associated toxic effects 52.

## 4. Isolation techniques

The purification of EVs is mandatory for most downstream analysis techniques. However, the separation is challenging due to the EVs' unique structure, complexity, and low concentration in complex biologic matrices. Among the different methods for EV isolation, differential and density gradient centrifugation, size exclusion chromatography, filtration, and polymer-based precipitation are the most popular.

Differential centrifugation remains the most commonly used technique for physically EVs isolation. The main objective of this method is to eliminate particulate interferents, including dead cells and debris, by successive centrifugation steps. An updated protocol with minor changes in centrifugation rate and times was recently proposed 53. In brief, four successive centrifugations are necessary to separate: 1) cells at 300 *g*; 2) dead cells and large cell debris at 2,000 *g*; 3) large and medium-sized EVs (or apoptotic bodies and microvesicles) at 10,000 *g*; and 4) small EVs (or exosomes) at 100,000 *g*. Differential centrifugation is a well-established and moderately time-consuming method that can be used with little or no sample pre-treatment. In addition, this approach is generally less expensive than density-gradient centrifugation and can provide high yields because EVs s are concentrated in the residual pellet, which contains fewer contaminants 54. In contrast, differential ultracentrifugation methods require costly equipment and can contribute to mechanical damage and low purity due to the co-existence of other non-EV vesicles and protein aggregates.

Size-exclusion chromatography (SEC) is a technique for separating particles based on their size. The interaction of the analysed particles with heterosporous polymeric beads results in hydrodynamic radius separation 55. Compared to individual proteins, protein aggregates, and other contaminants, the relatively larger size of EVs leads to high EV purity even from complex biologic matrices. Sepharose-based SEC columns are the most commonly used for EV isolation. For example, Sepharose CL-2B (Sigma-Aldrich Inc., St. Louis, MO, USA) can separate vesicles of 70 nm in diameter or larger. Although long elution times can occur, the mild conditions and reagent compatibility minimally affect EVs' biological activity, contributing to maintaining the integrity of the proteins and enzymes present in the EVs. The resulting high purity of these preparations enables their use for high-demanding “omics” downstream applications. The main shortcoming is related to the fact that the EVs are not preconcentrated by this strategy. It is important to note that commercial pre-packed Sepharose columns are specifically designed for EV isolation. These include Exopure™ (BioVision, Milpitas, CA, USA), PURE-EVs Columns (HansaBioMed, Tallinn, Estonia), and qEV Exosome Isolation (Izon Science, Christchurch, Canterbury, New Zealand).

As an alternative, microfiltration and ultrafiltration membranes can also be used for EVs isolation and, in some instances, in combination with ultracentrifugation and SEC. This approach is based on pore size filtration that allows particles with specific sizes or molecular weights (MW) to pass through while retaining larger-sized particles. Some commercial membranes are available in various pore sizes (800-100 nm). These include ExoMirTM (Bioo Scientific Corporation, Austin, TX, USA), Durapore® Membrane Filter (Merck Chemicals & Life Science S.A., Darmstadt, Hessen, Germany), Vivaspin® (Sartorius Lab Instruments GmbH & Co. KG, Goettingen, Lower Saxony, Germany), and the exoEasy Maxi Kit (Qiagen, Hilden, North Rhine-Westphalia, Germany).

Polymer-based precipitation relies on precipitation using polymers such as PEG 56. The principle of polymer-based precipitation is to use a reagent that binds to water molecules, thereby forcing the less-soluble components, such as EVs, to sediment, thus allowing collection by short, relatively low-speed centrifugation or filtration. Some commercial kits based on this approach include ExoQuick™ (System Biosciences, Mountain View, CA, USA), Exo-spin™ (Cell Guidance Systems, Babraham, Cambridge, UK), ExoPrep (HansaBioMed, Tallinn, Estonia), Exosome Purification Kit (Norgen Biotek, Thorold, Ontario, Canada), miRCURY Exosome Isolation Kit (Exiqon, Vedbaek, Denmark), and Total Exosome Isolation Reagent (Life Technologies Corporation, Carlsbad, CA, USA). Although costly, these polymer-based precipitation kits are user-friendly, rapid, and effective in isolating EVs in the 40-180 nm range while excluding larger EVs. Moreover, these kits have lower sample volume requirements, thus making them ideal for clinical use 57. However, this technique by itself is not useful for the isolation of EV from a specific cellular origin, since it is based on a non-specific polymer precipitation.

Immunomagnetic separation (IMS) uses biologically-modified magnetic particles (MPs) that can specifically bind targets. MPs are powerful and versatile separation and pre-concentration tools used in various analytical and biotechnology applications 58,59. MPs can be coated with antibodies to EV-receptor molecules. Once captured, a magnetic field is applied to separate the EV-coated MPs from the biologic matrix, thus significantly reducing matrix effects and contamination 60,61. Commercial superparamagnetic Dynabeads® M-450 Tosylactivated (Life Technologies Corporation, Carlsbad, CA, USA) (4.5 µm in diameter) are some of the MPs most commonly used to achieve this task. Other types of MPs are widely available from System Biosciences, Pierce, Miltenyi Biotec, HansaBioMed, Aethlon Medical, and New England Peptide, among others. Moreover, there are commercially available kits for separating EVs based on this principle, such as the MagCapture exosome isolation kit (Wako Life Sciences, Richmond, VA, USA). Likewise, several EV receptors have been used for IMS, including the most common tetraspanins CD9, CD63, and CD81 60,61. The main advantage of the IMS is the ability to isolate EVs by a specific immunological reaction, separating an EV subpopulation expressing a biomarker. Moreover, IMS is a very gentle approach that ensures both EV integrity and purity. It is easy to carry out by one-step incubation with the sample to form EV-magnetic particle complexes and does not require benchtop instrumentation. However, a remarkable disadvantage is that IMS is significantly more expensive than SEC, polymer-based precipitation, and filtration.

Finally, although EVs are commonly described to be isolated from body fluids and culture medium, there are also been described several methods to extract them from different tissues, including the brain. Most common method is based on a sucrose gradient ultracentrifugation of brain homogenates 62,63. Briefly, post-mortem tissues (previously stored at -80°C) are sliced lengthways on ice using a razor blade or cryostat (for human and mouse brains respectively) to generate wide sections of brain. The slices are homogenized and immersed in collagenase to dissociate the tissue. After a few cycles of centrifugations in PBS medium, the supernatant is overlaid on a triple sucrose cushion and ultracentrifuged during 3h at 180,000 × g (average) at 4°C. Obtained fractions are separated and diluted with ice-cold PBS and spun at 100,000 × g (average) at 4°C for 1 h to pellet the vesicles, and characterized to determine the nature of the vesicles. In addition, SEC is sometimes included after sucrose gradient to enhance the purity of obtained EVs fractions 64. The main advantage of this method, compared with previous methods in which tissue is subjected the tissue to filtering, blending and/or homogenization 65,66, or vortexing 67, is the enhancement of isolation enrichment, EVs purity and EV structure integrity preservation.

## 5. Biomedical applications of EVs

EVs have been considered as a molecular mirror of the cells they originate from and as potential targets for determining the state of cells in pathological conditions 68. Furthermore, they can be found in many biological fluids such as blood, CSF, urine, saliva, and milk 69, making them accessible biomarkers for complex diseases. Specifically, when focusing on neuronal diseases, the ability of EVs to cross the blood-brain barrier (BBB) makes them promising diagnostic and therapeutic biomarkers 70-72. However, there is little evidence about EVs crossing and there is needed more evidence to clarify the penetration process of EVs through the BBB.

Also, their role in intercellular communication opens an opportunity for their modification for therapeutic use.

### 5.1 The diagnostic capability of EVs

EVs are released into body fluids, allowing us to obtain representative information in biological fluids from less-accessible organs, as is the case of the central nervous system, during non-invasive procedures such as liquid biopsies. They reflect the cellular changes in pathobiology, even in the early stages. Because of this, the circulating EVs are a promising source of novel biomarkers for diagnosing and monitoring brain diseases. Some proteins are found to be ubiquitously useful as EV general markers 68,73,74. On the other hand, not only are proteins used as biomarkers but miRNA EVs expression patterns have also been reported to change in different brain diseases, such as AD and PD 75,76. These changes have been observed in different biofluids, such as CSF, serum and plasma 77.

Unfortunately, realizing the potential of these vesicles as biomarkers will require technical improvements since the EVs are exceptionally challenging to characterize with current technologies. EVs have a diameter of 30 to 200 nm, putting them outside the sensitivity range of most cell-oriented sorting or analysis platforms, such as classical flow cytometers. To date, the most common methods for targeting EVs have involved purification followed by specific characterization of their cargo 78. Isolation of the EVs is best performed with differential ultracentrifugation. Purification can also be done with precipitation, size-exclusion chromatography, or ultrafiltration 79. Lately, immunomagnetic separation is also used to isolate EV populations based on the universal membrane proteins, including CD9, CD63, or CD81 80, although as described above, they are not only specific of EVs' surface 14. Identification of membrane vesicles as EVs also requires morphological analysis 78. EVs can only be visualized with an electron microscope, given their small size. Nanoparticle tracking analysis (NTA) is usually used to count the EVs, followed by downstream processes for specific detection, including LC-MS/MS and Western Blot for proteins and qPCR for genetic material. The whole procedure is time-consuming and requires skilled personnel, laboratory facilities, and benchtop instrumentation. Current gold standard methodologies have limitations in isolating, detecting, and characterizing EVs with high specificity, sensitivity, and simplicity 56.

Numerous simplified analytical methods with various readouts have been reported recently for detecting EVs, including optical immunoassays 60, and electrochemical biosensors 61,81, among many others. In addition to their potential role as biomarkers, EVs are designed to carry molecular cargo from one cell to another 82. The EVs could thus be loaded with therapeutic cargo, enabling highly targeted delivery of drugs to specific types of cells while sparing all other cell types from damage 83.

### 5.2 The therapeutic potential of EVs

EVs are garnering considerable interest as promising candidates for therapeutic purposes due to their molecular-specific characteristics and carrying properties. In fact, EVs by themselves or as vehicles for the delivery of drug payload(s) are being actively explored as therapeutic agents. They are stable, biocompatible, can avoid the activation of the immune system, contain proteins that can prevent the activation of the coagulation cascade 84,85, and have been described to be able to cross blood vessels (including the BBB) 86, although this event must be deepen analyzed in further studies. All these characteristics confer them less toxicity and a lower clearance ratio compared to other therapeutic strategies. Moreover, EVs have cell tropism, allowing drug delivery specificity and are suitable for the transport of biological drugs such as proteins or nucleic acids (as short interference RNA (siRNA) or micro-RNA (miRNA)) 70,83. This nucleic acid cargo is also able to modify protein expression for therapeutic purposes. Besides these features, natural EVs are not enough for efficient therapeutic use, and they must be modified appropriately. Enrichment of EVs based on their surface ligand presentation may also enable the development of receptor-mediated tissue targeting 56. Pre-secretory drug loading and post-secretory drug loading are the two most common strategies for loading therapeutic cargo. In the pre-secretory way, the modification is performed in the cells that produce the EVs, while in the post-secretory way, modifications are performed directly on the EV surface or into its membrane (by electroporation, sonication, and other procedures) 56.

## 6. The role of EVs in neurological diseases

### 6.1 EVs in Alzheimer's disease

Dementia was the fifth-leading cause of death in 2016 87. AD is the most common form of dementia worldwide, constituting up to 50-80% of cases 87. AD is commonly diagnosed symptomatically through the occurrence of significant memory loss, global cognitive decline, and an overt impairment of daily life activities. Later in the course of the disease, the breakdown of physical functions, such as walking, swallowing, and general movement, ultimately leads to death 88. EVs have an important role in AD as they carry pathogenic proteins and spread them across the brain. Moreover, they are capable of inducing apoptosis of astrocytes by impairing neuronal function 89. EVs are involved in APP cleavage. Some proteases like BACE 1, PSEN 1, PSEN 2, and ADAM10 are present in MVB and exosomes. Furthermore, EVs can deliver mRNA that activates toll-like receptors, which upregulate the levels of proteases BACE1, PSEN1, and PSEN2 89. A PS gene mutation reduces Cystatin C (CysC) in EVs, which decreases soluble APP, and increases Aβ_1-42_ production 90. In this sense, Pérez-González *et al.* demonstrated that EVs containing CysC showed neuroprotection properties in cultured cells, as well as EVs secreted by CysC-deficient cells treated with exogenous human CysC significantly enhanced the survival of the cells 91. Other molecules participating in APP metabolisms, such as Grb2 or tetraspanin 6, have also been detected in EVs 77. Likewise, and study carried out with exosomes from human brain tissue showed that the exosome secretory pathway transports APP carboxyl-terminal fragments from the cell into the brain extracellular space 65. Moreover, ALIX, an EV marker, has been found in the senile plaques of AD brains 89. Astrocytes surrounding the senile plaques uptake Aβ peptide and release EVs with apoptotic proteins that lead to the apoptosis of astrocytes even if they have no contact with Aβ 77. However, EVs have also been described to possess neuroprotective effects in AD. The surface protein PrPc sequesters the Aβ oligomers, thus reducing the Aβ accumulation. Similarly, microglia can remove Aβ- EVs by recognizing nSMase2 and EV uptake, decreasing the extracellular Aβ levels 92. Microglia-derived exosomes (MDEs) also contribute to spreading pathogenic tau 89. Interestingly, mouse tauopathy models that have depleted microglia have shown an inhibition of tau propagation 92. The genotype has also been related to EVs in AD. A study conducted in n human postmortem tissues and humanized mouse models for apolipoprotein E showed that the apolipoprotein E4 genotype leads to downregulation of exosome biosynthesis and release 93.

Many studies are currently evaluating the potential of EVs as diagnostic and therapeutic tools in AD (**Table 1**). In that sense, *Goetzl et al.* explored the functionally specialized synaptic protein cargo of plasma neuronal-derived exosomes (NDEs) of patients with AD dementia and frontotemporal dementia (FTD) 94. One of the main molecular changes in the early stages of AD is the altered synaptic protein levels 95,96. They found that four synaptic proteins (synaptophysin, synaptopodin, synaptotagmin, and neurogranin) were significantly decreased not just in AD patients but also in FTD patients. Moreover, the serine 9 phosphorylation of synapsin 1 was reduced in AD and FTD, indicating that the protein's functionality is also altered. Only AD patients exhibited a significant reduction in GAP-43 and synapsin 1 levels. Taking these together, the authors suggest that NDE synaptic proteins may be useful for preclinical dementia detection and disease progression 94. The same authors found that protein levels of presynaptic proteins NPTX2, NRXN2α, and their postsynaptic partners AMP4 and NLGN1 were significantly decreased in plasma NDEs and correlated with cognitive loss 97. Additionally, except for NPTX2, these proteins were found to be reduced even in preclinical AD patients, and all of them declined with AD progression 97.

Liu *et al.* also studied the potential of the synaptic protein neurogranin (Ng) as a neurodegeneration biomarker in plasma exosomes 98. In recent years, clinical studies have reported that CSF and blood Ng levels are closely related to the occurrence and subsequent progression of AD 99-101. Thus, the authors conducted a meta-analysis including 4661 individuals, of which 187 were selected for exosomes analysis. Ng levels were found to increase in CSF but decrease in plasma exosomes of patients with AD and mild cognitive impairment (MCI)-AD and to be highly associated with cognitive decline. Interestingly, the concentration of Ng in plasma exosomes of AD and MCI patients was lower than that of healthy controls. Moreover, this concentration was also lower in MCI-AD patients than in those with a stable MCI diagnosis 98. Nielsen *et al.* evaluated the protein cargo of plasma-derived EVs related to neurological and inflammatory processes in patients with AD, MCI, and healthy controls 102. They analyzed 182 proteins by using the proximity extension assay technology of Olink proteomics 103-105. Although no group differences in particle concentration or size were found in EVs samples, the CLEC1B/CCL11 ratio showed diagnostic capabilities between healthy controls and AD. Similarly, Winston* et al.* predicted conversion from MCI to dementia through the study of plasma NDE protein profiles 106. In this study, the authors also found that Ng levels were significantly lower in AD and MCI-AD compared to cognitively normal controls. In contrast, the levels of p-tau^T181^, p-tau^S396^, and Aβ_1-42_ in plasma NDEs were significantly higher than those in AD NDEs. In addition, the authors proved that NDEs contribute to AD pathology by injecting plasma NDEs from AD and MCI patients into wild-type mice. The results showed that mice injected with AD NDEs had more p-tau positive cells in the CA1 region of the hippocampus than those littermates injected with MCI NDEs 106.

In a later study, the authors also evaluated the progression from MCI to AD dementia by analyzing the abnormalities of complement proteins (CPs) in plasma astrocyte-derived exosomes (ADEs) 107. Several pieces of evidence have suggested that complement system proteins may mediate the capacity of type A1 astrocytes to damage neurons 108-110. Moreover, astrocytes expressing type A1 markers in areas of postmortem brain tissues affected by neuroinflammatory or neurodegenerative diseases have high levels of complement component 3 (C3) 111. In this case, ADEs showed significantly increased levels of some proteins from both the classical and alternative complement pathways. The CD46, CD59, and type 1 complement receptor levels were significantly lower in MCI converting patients than in stable MCI patients 107. The authors theorized that the increased C3b opsonization by neurons might be one of the causes of the attraction and neuronal toxicity mediated by microglia, while the high levels of the complex C5b-C9 might cause direct injury to neurons. However, further studies are needed to clarify which CPs could distinguish between stable and converting MCIs patients, their contribution to neurotoxic neuroinflammation, and their potential as predictive biomarkers 107.

The same authors evaluated the biomarker profile variations in plasma NDEs from participants enrolled in a randomized, double-blind, placebo-controlled 20-week trial of growth hormone-releasing hormone (GHRH) administration 112. The beneficial effects of insulin growth factor 1 (IGF-1), whose release is stimulated by GHRH in physiological conditions, on brain health are well described, as is their reduction caused by aging, which has been associated with the development of AD 113-115. The authors found that concentrations of Ng, synaptophysin, synaptotagmin, and synaptopodin were significantly decreased in NDEs of patients with MCI, whereas Aβ_1-42_ was significantly increased, independent of GHRH treatment. On the other hand, p-tau^S396^ and GAP43 were not affected by cognitive status or GHRH treatment. Thus, even though NDEs biomarkers cargo showed promising results, the treatment with GHRH did not clarify its role in the clearing mechanisms associated with reducing AD neuropathology in the brain 112. Similarly, Mustapic *et al.* aimed to study the neuroprotection effect of insulin in the brain through the analysis of exosomes protein cargo. Thus, they measured the levels of pS312-IRS-1 and pY-IRS-1, biomarkers of insulin resistance, in NDEs to track the response of AD and MCI patients to intranasal insulin treatment 116. They found differences in both biomarkers in response to the 20 IU treatment but not for the placebo or 40 IU. Interestingly, these changes were also correlated with cognition scores 116.

The study of the variabilities in the miRNA profile of the EVs is another of the most interesting approaches to this type of study. In this regard, Yang *et al.* evaluated the expression levels of exosomes miRNA-135a, miRNA-384, and miRNA-193b, which were observed to be involved in the regulation of APP and BACE1 in AD, vascular dementia (VD), and Parkinson's disease patients 117. They found that miRNA-135a and miRNA-384 were upregulated while miRNA-193b was downregulated. The best candidate for differentiating between AD, VD, and PD was mi-RNA-384, while the combination of all three was proved to be better than a particular one for early AD diagnosis 117.

Liu *et al.* also studied the diagnostic potential of miRNA-135a and miRNA-193b loaded into ATP-binding cassette transporter ABCA1-labeled exosomes in both preclinical and clinical samples of AD 118. ABCA1 mediates cellular cholesterol efflux in the brain and participates in neuroinflammation and neurodegeneration processes 119. The authors found that ABCA1-labeled exosomes containing miRNA-135a were able to cross the BBB and were increased in the CSF and serum of transgenic mice. In addition, ABCA1-labeled exosomal content of miR-135a was significantly increased in the CSF and serum of MCI and AD patients compared to those of the control group, agreeing with Yang *et al.'*s findings 118. In the study of miRNA-193b, the authors found that wild-type mice injected with ABCA1-exosomes from the CSF of APP/PS1 mice exhibited increased levels of miRNA-193b in both CSF and serum. As with miRNA-135-a, ABCA1-labeled exosomal miRNA-193b levels were also higher in both CSF and serum of MCI and AD patients compared with the control group 120. However, in this case, miRNA-193b appeared to be up-regulated in ABCA1-labeled exosomes of AD patients contradicting the results found by Yang* et al.*

Other fluids have also been studied as a source of EVs. Moreover, the concentration of EV protein cargo per se has also been reported as a differential parameter of disease progression 121. In that sense, Rani* et al*. developed a new method based on nanoparticle tracking analysis to purify salivary exosomes from patients with AD, MCI, and VD**.** Using their method, the authors observed significant differentiation in salivary exosomal concentrations. Specifically, exosomes concentrations were found to be significantly higher in AD and MCI compared to controls, while they were slightly higher between AD and MCI groups 122. Moreover, pathological proteins (Aβ, p-tau, and Aβ oligomers/fibrils) found in exosomes of both AD and MCI were significantly higher than in control groups.

### 6.2 EVs in Parkinson's disease

PD is characterized by a progressive loss of dopaminergic neurons, mainly present in the substantia nigra, accompanied by the degeneration of dopaminergic terminals in the striatum, thus leading to movement coordination impairments and cognitive decline, depression, or anxiety. It is the most common motor disorder and the second most prevalent neurodegenerative disease. It is incurable, with an unknown etiology in most cases. A systematic analysis for the *Global Burden of Disease Study 2016* pointed out that PD was the disease with the fastest-growing prevalence, disability, and death rates, affecting 1% of the population over the age of 60, or 1-2 individuals over the age of 60 per 1000 people at any given time 123.

EVs have been observed to contain α-synuclein, the essential protein involved in the pathogenesis of PD. Acceleration of α-synuclein accumulation 124 and cytotoxic effects have been associated with α-synuclein EV uptake 125. EVs are involved in transmitting α-synuclein between cells, facilitating its propagation 126 and the pathogenesis severity 125. Interestingly, some PD genes such as LRRK 2, VPS 35 and PARK 9 are related to the autophagic-lysosomal pathway of α-synuclein clearance 125. This pathway is inhibited or at least dysfunctional in PD subjects, thus leading to impaired protein clearance. This alteration increases EV release to avoid intracellular α-synuclein accumulation but enhances extracellular accumulation and the spread of PD pathophysiology 126. In fact, the pathological mutation of LRRK2 resulted in an increase in the number of EVs due to an abnormal function of MVBs. Furthermore, it has been reported that the levels of PARK9 and EVs are positively correlated, thus suggesting that α-synuclein extracellular levels are modulated by PARK9 through EV secretion 125.

Several studies have evaluated the diagnostic potential of EVs in PD and their role in disease spreading (**Table 2**). In that regard, Shi *et al.* evaluated the performance of blood α-synuclein as a PD biomarker through its analysis in plasma exosomes 127. Previous studies reported plasma α-synuclein to be inconsistent and generally ineffective for PD diagnosis 128. Preclinical *in vivo* findings showed that the α-synuclein could be transported from the brain to the peripheral blood into exosomes. Furthermore, they found that the patients with PD presented a higher concentration of α-synuclein in L1CAM-enriched plasma exosomes, which was significantly correlated with the disease severity. Although further studies are needed to clarify the physiopathology molecular mechanisms, the authors suggest that this increased efflux of α-synuclein into exosomes is a cleavage mechanism to reduce the α-synuclein central toxicity 127. About L1CAM, this transmembrane protein is expressed on neurons and widely used to isolate neuron-derived EVs (NDEVs) in human biofluids. However, there is much controversy about the utility of L1CAM to track neuronal origin exosomes. A recent study developed by Norman *et al.* aimed to understand why there was observed increased levels of α synuclein in NDEVs samples isolated by capture with an anti-L1CAM antibody 129. They showed that this increase were not related to EVs of plasma or CSF, but a nonspecific binding of soluble α-synuclein (and other soluble proteins) to the anti-L1CAM antibody 129. In this sense, further studies are needed to find a real specific marker of NDEVs and highlighting the importance of performing rigorous controls to exclude these false positives.

Zheng *et al.* investigated α-synuclein species, oligomeric and phosphorylated α-synuclein in plasma exosomes as potential PD peripheral biomarkers 130. The authors found that aggregated α-synuclein and p-α-synuclein existed inside and on plasma exosomes' membrane surfaces. In addition, the ratio of p-α-synuclein oligomer/total p-α-synuclein was shown to be moderately helpful in PD diagnosis 130.

The utility of EVs is not only restricted to diagnosis purposes but also monitoring and treatment of PD. In this sense, Athauda *et al.* performed a proteomic assay in plasma exosomes of patients enrolled in a randomized, placebo-controlled clinical trial to assess the influence of Exenatide treatment on brain insulin and protein activity kinase B (Akt) signaling pathways 131. Exenatide, a glucagon-like peptide 1 agonist used in type 2 diabetes, was recently found to have beneficial effects on the motor function of PD 132. In this study, the authors found that NDEs of treated patients exhibited elevated expression of downstream substrates, including total Akt and phosphorylated mechanistic target of rapamycin (mTOR), as well as augmented tyrosine phosphorylation of insulin receptor substrate compared to the placebo group 131. These results highlighted the potential of using EVs -based biomarkers as monitoring objective measures for clinical trials.

Blood EVs carrying miRNAs have been highlighted to serve as potential targets for diagnosis and PD treatment. In that sense, Jiang *et al.* aimed to demonstrate in a preclinical study that exosomal miRNA-137 isolated from serum might have an effect on PD by regulating oxidation resistance 1 (OXR1), a protein involved in oxidative stress-induced neurodegeneration 133. These authors found that the inhibition of miRNA-137 or up-regulation of OXR1 ameliorated PD-induced oxidative stress injury. Furthermore, when exosomal miRNA-137 was inhibited using miR-137 antagomir, an amelioration of PD-induced oxidative stress injury was observed in an *in vitro* model. Thus, the down-regulation of exosomal miR-137 alleviates oxidative stress injury in PD by upregulating OXR1 133.

Li *et al.* explored the therapeutic potential of exosomes by suppressing the autophagy processes in the dopaminergic neurons of the *substantia nigra* 134. In this case, exosomes serve as genetic vectors for miRNA-188-3p, which is found to be an autophagy and pyroptosis suppressor by targeting CDK5 and NLRP3 135-137. They assessed the levels of autophagy, injury, and inflammasomes in PD mouse and cell models and found that these processes were suppressed in both models after treating them with miRNA-188-3p-enriched exosomes 134. Yang *et al.* also investigated the use of exosomes as carriers of antisense oligonucleotides (ASO) to reduce α-synuclein expression in PD 138. Knockdown ASO-based gene strategy is a reliable and well-established method for treating neurodegenerative diseases, such as PD 139. The authors found that ASO loaded into exosomes showed high cellular uptake and low toxicity in primary neuronal cultures. Furthermore, in an α-synuclein A53T transgenic mouse PD model, intracerebroventricular injection of exo-ASO significantly decreased the expression of α-synuclein and attenuated its aggregation, ameliorating the degeneration of dopaminergic neurons and improving the locomotor functions of treated mice 138.

### 6.3 EVs in multiple sclerosis

Multiple sclerosis (MS) is the most common inflammatory neurological disease in young adults. It is a chronic, heterogeneous, disabling autoimmune disease characterized by demyelination, neuroinflammation, oligodendropathy and neuronal degeneration that affects both gray and white matter 140. Although the exact etiology of MS is not clear yet, demyelination is the main alteration found in these patients. In addition, EVs have a vital role in CNS myelination development 141. Oligodendrocytes secrete EVs that slow the formation of the myelin sheath, while neuronal EVs s inhibit oligodendrocyte-derived EVs. Therefore, EVs contribute to the homeostasis of myelin biogenesis 142. Moreover, it has been reported that EVs, released by mature oligodendrocytes activated with glutamate through the NMDA and AMPA pathway, stimulate the transduction of myelin proteins and the promotion of specific RNA 143. However, EVs have also been shown to negatively contribute to the demyelination process. Endothelial cells, leucocytes, microglia, astrocytes, and platelets can be released in response to proinflammatory cytokines. EVs carrying metalloproteinases and caspase 1 can induce disintegration of the BBB and facilitate lymphocyte and myeloid cell transmigration into the CNS 144. Furthermore, endothelial cells transfer integrin Mac 1 and ICAM 1 receptors to monocytes through EVs, which enhances their ability to cross the endothelial barrier 145. Collectively, they facilitate the transmigration of lymphocyte cells into the brain and the onset of the autoimmune response typical of MS 141.

Based on this evidence, most studies have deepened the potential of EVs in MS (**Table 3**). Ebrahimkhani *et al.* explored the exosomal miRNA signatures as monitoring and diagnostic tools for MS 146. When comparing the exosome-associated microRNAs in serum samples of MS patients and healthy controls, they found several differentially expressed exosomal miRNAs in both relapsing-remitting MS and progressive MS patients compared to controls. In addition, nine of these miRNAs showed significant scores when validated in an independent group of progressive MS cases 146.

In an interesting study by Mrad *et al.*, they compared the effects of serum-derived exosomes on monocyte-derived macrophages (MDMs) of healthy donors and those of patients with relapsing-remitting MS in remission and relapse 147. The main purpose of this study was to assess whether the response correlates with exosomal Epstein-Barr virus (EBV) protein expression. EBV has been highly associated with MS 148. In fact, scientific evidence has shown that most MS patients are infected with EBV and have increasing titers of antibodies against the EBV nuclear antigen EBNA1 before the onset of neurologic symptoms 149,150. The results found in this study revealed that the expression of latent membrane proteins LMP1 and 2A and EBV nuclear antigen EBNA1 was higher on exosomes derived from patients with active MS compared with healthy controls and stable patients. Moreover, MDMs differentiated from patients with active disease were better secretors of CXCL10, CCL2 and CXCL9, than MDMs from healthy and stable MS groups 147. Thus, the authors suggest that EBV(+) exosomes could promote a proinflammatory response in MS patients by antigen-presenting cells and may contribute to ongoing immune activation.

Several authors have studied the therapeutic potential of EVs in MS against the demyelination process by targeting the neuroinflammation cascade. In that sense, Li *et al.* explored the use of exosomes secreted by bone marrow mesenchymal stem cells (BMSCs) in the experimental autoimmune encephalomyelitis (EAE) rat model of MS 151. Recent studies have shown that BMSC exosomes play a crucial role in many autoimmune diseases and aid in tissue repair, but the specific mechanisms are still unknown 152,153. These authors discovered that exosome treatment significantly reduced inflammatory cell infiltration into the CNS, neural behavioral scores, TGF-α and IL-12 levels, and the demyelination process. In contrast, mRNA expression levels of M2 phenotype markers IL-10 and TGF-β were significantly increased. Similarly, Fathollahi *et al.* evaluated the therapeutic potential of MSC-derived exosomes in an EAE mouse model of MS. In this case, the aim of the study was to evaluate, using clinical symptoms and histological analysis of CNS lesions, the effect of intranasal administration of SEV on disease activity and antigen-specific responses 154. The results showed that treatment with MSC-exosomes significantly decreased clinical scores and was more effective in alleviating clinical scores than MSC alone. In addition, this decrease was associated with an increase in immunomodulatory responses, such as Foxp3, CD25, and regulatory T cell frequency. In this regard, Hosseini Shamili *et al.* also evaluated the immunomodulatory properties of MSC-derived exosomes as a platform for reducing MS clinical scores 155. In this case, the LJM-3064 aptamer, which has been shown to possess a specific affinity toward myelin and has demonstrated re-myelination properties 156, was covalently conjugated to the surface of the exosomes and employed as both a targeting ligand and a therapeutic agent. Their results showed that exosomes promote the proliferation of oligodendroglia in the in vitro assays and reduce the inflammatory response and demyelination in the in vivo studies 155. Finally, Pusic *et al.* conducted an interesting study in which dendritic cell cultures were stimulated with low-level IFNγ exosomes 157. These exosomes contained miRNAs that could increase baseline myelination, reduce oxidative stress, and improve re-myelination processes. The results also showed that these exosomes were preferentially taken up by oligodendrocytes, thus suggesting that they could positively affect myelination 157. These results exhibit great potential for the use of these EVs as a re-myelination therapeutic tool not only in multiple sclerosis but also in other dys-myelinating syndromes.

### 6.4 EVs in amyotrophic lateral sclerosis

ALS is a fatal motor neuron degenerative disease with high socioeconomic impact, affecting approximately 300,000 patients worldwide and expected to increase to 400,000 by 2040 158,159. The etiology of the disease is still unknown, but several proteins have been identified to be involved in its pathogenesis. Some of these proteins, such as SOD1, p-TDP-43, or FUS, have been shown to be transported from cell to cell by the EVs pathway, contributing to the propagation of prion-like misfolded isoforms and increasing the neuropathology of ALS 160. Likewise, the levels of IL-6 have been shown to be increased in astrocyte-derived EVs and are positively correlated with disease progression 160. Similarly, EVs-driven miRNAs have been shown to be altered in ALS patients, with fewer reads than healthy subjects and different expression patterns 161.

In this sense, many research groups are currently evaluating the potential of EVs as diagnostic and therapeutic tools in ALS (**Table 4**). Basso *et al.* explored the influence of mutant SOD on the protein secretion pathway and exosome release in astrocytes and its implications for disease spread and motor neuron pathology 162. SOD1 mutations are the major genetic contributors to ALS, with more than 170 SOD1 mutations identified 163. The obtained results showed that mutant SOD1 astrocytes released a larger proportion of proteins through exosome spreading. Moreover, the authors found that astrocyte-derived exosomes transferred SOD1 to spinal neurons and induced selective motor neuronal death 162. Thus, these findings suggest that astrocyte-derived exosomes may have a role in disease spreading and motor neuron pathology. New therapeutic approaches could target exosomes to contain disease progression. Silverman *et al.* also explored the origin of CNS-derived extracellular vesicles carrying misfolded SOD1 in a mouse model of ALS 164. In this case, quantitative proteomics comparing brain-derived EVs from non-transgenic and transgenic ALS mice (SOD1G93A mice) revealed that these EVs contain canonical exosomal markers enriched in synaptic and RNA-binding proteins. The complete brain EV proteome contained various proteins implicated in ALS, especially in EVs from SOD1G93A mice, which were significantly depleted in myelin oligodendrocyte glycoprotein compared with those from non-transgenic mice 164. They observed that brain- and spinal cord-derived EVs from both NTg mouse models were positive for the astrocyte marker GLAST and the synaptic marker SNAP25, whereas the microglial marker CD11b was largely absent.

Moreover, EVs from the brains and spinal cords of the SOD1G93A ALS mice and human SOD1 familial ALS patients' spinal cords contained abundant misfolded SOD1 164. These results suggest that brain astrocytes and neurons, but not microglia, are the main EV sources and are involved in disease development through their pathogenic disease-causing proteins.

Bonafede *et al.* investigated the neuroprotective properties of exosomes in several in vitro models of ALS. In their first work, the authors hypothesized that the administration of exosomes from adipose-derived stromal cells (ASCs) could ameliorate ALS symptoms since ASCs have been demonstrated to promote neuroprotection and neurodegeneration. For this purpose, they used NSC-34 cells transfected with different human mutant SOD1 transgenes, which are commonly used to mimic a motoneuron-like behavior in ALS 165. Obtained results showed that, in both naïve and over expressing mutant SOD1 NSC-34 cells, the addition of ACS-derived exosomes rescued cells from H_2_O_2_ oxidative stress-induced death 165. In a later study, the same authors evaluated the influence of ASC-derived exosomes on disease progression in a SOD1G93A ALS mouse model 166. In this case, exosomes were administered intranasally and intravenously, and their effects were tested on glial cells, lumbar motoneurons, neuromuscular junction, and muscle. Their results showed that ASC-exosomes protected muscle, lumbar motoneurons, and the neuromuscular junction, which resulted in improved motor performance 166. Likewise, a decrease in glial cell activation was observed in treated SOD1G93A mice. Curiously, exosomes were found to target lesioned ALS regions of the brain. These results together highlight the potential of exosomes as a novel treatment strategy for this incurable disease.

Regarding diagnostic purposes, Chen *et al.* conducted a longitudinal clinical trial in which 18 ALS patients were recruited, plasma samples were extracted at different time points, and exosomes were isolated by immunoprecipitation 167. Neurofilament light chain (NfL), phosphorylated neurofilament heavy chain (pNfH) by ELISA, and exosomal TAR DNA-binding protein-43 (TDP-43) ratio were selected as the study biomarkers. TDP-43 is the major component of the ubiquitinated inclusions that appear in the brains of 95%-97% of ALS patients 168,169. Their results showed that the exosomal TDP-43 ratio significantly changed in 3- and 6-month follow-up and that NfL (but not pNfH) was significantly higher in the rapid progression group at baseline and 3-month follow-up 167. This suggests that plasma NfL and exosomal TDP-43 ratio could be potential biomarkers for ALS long-term follow-up studies. Similarly, Hayashi *et al.* developed a clinical trial in which a proteomic analysis of exosome-enriched fractions derived from the CSF of amyotrophic lateral sclerosis patients was performed 170. They used liquid chromatography-tandem mass spectrometry (LC-MS/MS) for the proteomic analysis, and a wide variety of proteins were compared to idiopathic normal-pressure hydrocephalus (iNPH) patients as disease controls. Their results revealed that a novel INHAT repressor (NIR) was increased in exosome-enriched fractions of the CSF. Likewise, these findings confirmed that NIR expression was reduced in motor neurons of sporadic ALS patients, thus suggesting NIR as a candidate biomarker protein for ALS 170. Other techniques have been explored to analyze the protein content of EVs in ALS patients. Thus, in a recent study by Morasso *et al.*, Raman spectroscopy was used to evaluate the molecular differences in plasma-derived EVs between sporadic ALS patients and sex- and age-matched healthy controls 171. In this case, Raman spectroscopy provided an overview of the biochemical composition of EVs. The EVs of ALS patients exhibited a pattern of Raman spectra that was distinct from that of the controls. Moreover, amino acids like tryptophan or carotenoids showed different intensities of selected peaks in the spectra of both groups of patients in the EV compartment 171.

The study of miRNA content in EVs is also of interest in ALS research for diagnosis purposes. In this sense, Saucier *et al.* conducted a study to identify an ALS-associated miRNA signature in EVs, which can cross the BBB and enter the circulatory system, obtained from plasma samples of people diagnosed with ALS 172. Next-generation sequencing was used to identify differentially expressed miRNAs recovered from EVs of ALS patients and healthy controls. Their results revealed elevated levels of 5 miRNAs and reduced levels of 22 miRNAs in the EVs of ALS patients compared with healthy controls. Moreover, miRNAs with relevance to ALS were found to be deregulated. A few miRNAs were also identified as potential diagnostic tools for their association with disability progression in ALS. In addition, functional assessment of transcripts targeted by select ALS-associated miRNAs revealed processes such as transcriptional regulation and protein ubiquitination in these patients 172. All these findings highlight the miRNA signature in EVs as a potential diagnostic strategy for this condition.

### 6.5 EVs in brain cancer

Brain tumors are one of the most feared cancer forms and are responsible for substantial morbidity and mortality worldwide. Their incidence has significantly increased in the last three decades 173. The last Global Burden of Disease report indicated 330,000 incident cases of CNS cancer and 227,000 deaths globally 174. Moreover, it was estimated that the incidence rate of all brain tumors was 10.82 per 100,000 person-years 175. Glioblastoma multiforme (GBM) is the most common malignant primary brain tumor. This form comprises 27% of all tumors, and 80% of malignant tumors 173. It is estimated that more than two-thirds of adults diagnosed with glioblastoma will die within 2 years of diagnosis 176. Gliomas are primary brain cancers originating from progenitor cells, neural stem cells, or differentiated mature neural cells that are transformed into tumor stem cells 177. In turn, they can be classified according to their aggressiveness, ranging from 2 to 4, or according to their origin, which could be oligodendroglioma, astrocytoma, or oligoastrocytoma.

A growing body of evidence suggests that gliomas can influence cells both nearby and beyond the BBB through the regulated excretion of EVs 178. In GBMs, the EV content reflects the tumor's unique signature of EGFRvIII mutation, EGFR amplification, TGF-β, IDH1 mutation R132H and podoplanin status 7. In addition, EVs are also implicated in the transfection of receptors such as PDGFR and HER 2 to cells that do not have them, thus contributing to tumor proliferation. Interestingly, glioma stem cells are able to resist chemotherapy and transfer some resistance characteristics through EVs, for instance, the surface markers CD133 and CD44 or the adenosine-producing enzyme, which is a cell receptor responsible for the cells' drug tolerance 179. Furthermore, GBM-derived EVs have also been described to induce angiogenesis during hypoxia by delivery of proteins (such as IL-8, MMP 8, or PDGF) and mRNA transcripts to pericytes and vascular endothelial cells 180. Moreover, not only in GBM but also in other tumors, EVs have also been shown to contribute to immunosuppression. Notably, serum EVs from patients with advanced melanoma or colon carcinoma inhibited monocyte precursor differentiation into dendritic cells and programmed the precursors to become myeloid suppressive cells, which suppress T lymphocytes through TGF-β secretion, down-regulation of surface MHC class II expression, and persistent CD14 pattern recognition receptor positivity. In contrast, EVs derived from healthy controls encouraged T lymphocyte stimulation and myeloid maturation 181.

Many authors have studied the potential of EVs as diagnostic tools in GBM and their role in disease spreading (**Table 5**). Xu *et al.* investigated the effects of glioma-derived exosomes on autophagy, tumor-associated macrophages (TAMs) polarization, and glioma progression in the human GBM cell lines U87MG and U251 and the human monocyte cell lines U937 and THP-1. Subsequently, they evaluated the roles of hypoxic glioma-derived exosomes (H-GDEs) *in vivo* through tumor xenografts in a BALB/c nude mouse model 182. Their results indicated that H-GDEs markedly facilitated autophagy and M2-like macrophage polarization compared to normoxic glioma-derived exosomes (N-GDEs). Furthermore, IL-6 and miR-155-3p were highly expressed in H-GDEs and induced M2-like macrophage polarization through the IL-6-pSTAT3-miR-155-3p-autophagy-pSTAT3 positive feedback loop. These alterations promoted glioma proliferation and migration *in vitro* and *in vivo*. These findings suggest IL-6 and miR-155-3p as novel biomarkers for diagnosis and antitumor immunotherapy of GBM 182. Iorgulescu* et al.* also explored the immunosuppressive properties of exosomes in the established glioma cell lines U87 and U251. Primary GBM lines, obtained from fresh tumor tissue of two patients, were also included in the study 183. Results showed that GDEs displayed exosome-specific markers, but failed to recapitulate the antigen-presentation machinery, surface co-modulatory signals, or immunosuppressive mediator status of their parent tumor cells. This suggests that GDEs are restricted in their capacity to directly prime peripheral immunosuppression 183.

The role of EVs miRNAs on tumor growth and metastasis has gained interest in recent years. Cai *et al.* tried to elucidate the mechanisms through which exosomal miRNAs contribute to GBM cell metastasis by analyzing the effects of exosomes from 30 GBM patients and 30 healthy volunteers in the human glioblastoma cell line T98G and the human kidney cell line HEK 184. They identified miR-148a as an exosomal risk factor for metastasis. Results showed that circulating exosomal miR-148a levels were significantly higher in serum from GBM patients compared with serum from healthy volunteers. In addition, they also found that both protein and mRNA levels of cell adhesion molecule 1 (CADM1) were decreased in the tissues of GBM patients. In T98G cells, inhibition of miR-148a suppressed cell proliferation and metastasis. Interestingly, there was a strong negative correlation between exosomal miR-148a and CADM1 mRNA levels in samples of patients 184. These findings suggest that exosomal miR-148a could be a potential treatment target against proliferation and metastasis in GBM patients.

GBM is one of the most highly vascularized human cancers. As previously stated, the role of EVs in cancer angiogenesis has recently gained attention, but the proangiogenic molecules carried within have yet to be identified. In this regard, Wang *et al.* investigated the role of vascular endothelial growth factor (VEGF) in GDEs in GBM cell lines and primary cultures 185. Their findings revealed that VEGF-C from GDEs, by binding to VEGF receptor 2, stimulated Tafazzin expression in endothelial cells (a mitochondrial enzyme that exchanges fatty acids between phospholipids) 186. This event is derived from stimulating endothelial cell viability, migration, and tubulation. In addition, they demonstrated that the inhibition of exosomal release, together with bevacizumab treatment, had a combined inhibitory effect on angiogenesis 185. Similarly, Li *et al.* studied the miRNA signatures in exosomes to evaluate the effect of radiotherapy in glioma patients 187. Exosomes were isolated before and after radiotherapy, and exosome-derived miRNA sequencing was performed. A total of 18 upregulated differentially expressed miRNAs and 16 down-regulated differentially expressed miRNAs were identified. Following radiotherapy, qPCR analysis revealed that the hsa-miR-6731-5p and hsa-miR-208b-3p were overexpressed, while the hsa-miR-2116-3p was underexpressed. Moreover, target genes of identified miRNAs were shown to be primarily involved in metabolic processes, p53 signaling pathways, and cancer pathways. This suggests that these miRNAs could play a crucial role in glioma development 187.

Ding *et al.* evaluated the role of circular RNAs (circRNA) in the resistance to chemotherapy treatments, a major obstacle for glioma management 188. circRNAs are a novel class of endogenous non-coding RNAs that form covalently closed loops 189. These molecules have been found to be associated with the initiation and progression of different human diseases, including cancers 190. In this work, the authors explored the effect and underlying mechanism of exosomal circRNA nuclear factor I X (CircNFIX) on temozolomide (TMZ) chemoresistance in glioma. Results showed that exosomal CircNFIX conferred TMZ resistance to recipient sensitive cells through the enhancement of cell migration, invasion and repression of cell apoptosis under TMZ exposure. Exosomal CircNFIX also promoted tumor growth and its depletion enhanced TMZ sensitivity in glioma cells in vivo. Furthermore, the authors found that CircNFIX directly interacted with miR-132, whereas CircNFIX knockdown enhanced TMZ sensitivity in resistant glioma cells by up-regulating miR-132 188. This suggests that exosome-mediated transfer of CircNFIX partially enhances TMZ resistance in glioma through the miR-132 pathway, thus highlighting a potential prognostic biomarker for improving the clinical benefits of TMZ treatment. Wang *et al.* also explored the influence of exosomes in chemoresistance in gliomas, but in this case, in terms of improving the efficacy of drugs. Based on the intrinsic inflammatory chemotaxis and excellent BBB-crossing capability of neutrophils, the authors designed a bioinspired neutrophil-exosomes (NEs-Exos) system with loaded doxorubicin (DOX) and evaluated it in two *in vivo* models of zebrafish and C6-Luc glioma-bearing mice 191. In the first step, the authors extracted NEs from the bone marrow of mice. Subsequently, exosomes were isolated from these NEs. Their results showed that NEs-Exos rapidly penetrated the BBB and migrated into the brain. Additionally, NEs-Exos responded chemotactically to inflammatory stimuli and targeted infiltrating tumor cells. Moreover, DOX-NEs-Exos efficiently suppressed tumor growth and prolonged survival time in glioma *in vivo* models 191. These results represent a promising chemotherapeutic approach for the clinical treatment of glioma and other brain diseases.

## 7. Conclusion

The biogenesis of EVs is initiated by an invagination of the plasma membrane of the origin cell. This invagination includes a cell-surface protein-specific fingerprint and soluble molecules associated with the extracellular matrix, such as ions, metabolites, peptides, or lipids. This gives EVs a crucial role as promising biomarker tools for many diseases, in addition to their biological relevance and easy accessibility from a broad range of body fluids. They have been identified in various human diseases, such as AD, cancers, cardiovascular diseases, sarcoidosis, or prion diseases. In addition, they have been described to be involved in many physiological functions, such as cellular intercommunication, maintenance and improvement of synaptic plasticity, neurotransmission in the adult brain, innate immune responses, human reproduction, pregnancy, and embryonic development or wound healing. Since EVs are released into body fluids, this affords us the opportunity to use these vesicles as promising candidates for diagnostic and therapeutic purposes. Specifically, they have been shown to possess critical information about pathological processes underlying major brain diseases. In AD, it has been described that EVs play an important role in spreading hallmarks across the brain and are involved in APP cleavage, delivery of pathogenic mRNA, and an increase in Aβ_1-42_ production. In PD, EVs have been shown to contain α-synuclein, which has been related to the acceleration of the α-synuclein cytotoxic effects and the pathogenesis severity. In MS, EVs secreted by oligodendrocytes negatively contribute to the homeostasis of myelin biogenesis and induce disintegration of the BBB, thus facilitating lymphocyte and myeloid cell transmigration into the CNS. In ALS, several proteins involved in its pathogenesis have been shown to be transported from cell to cell by the EV pathway, contributing to the propagation of prion-like misfolded isoforms and increasing the neuropathology of ALS. Finally, in GBM, a growing body of evidence suggests that gliomas can influence cells both nearby and beyond the BBB through the regulated excretion of EVs. The EV content of GBMs reflects the tumor's unique signature and is implicated in the transfection of receptors such as PDGFR and HER2 to non-tumoral cells. In conclusion, EVs have a promising future as drug targets, monitoring tools, and diagnostic tools for many diseases, especially those with difficult access and complex etiologies, such as major brain diseases.

## Figures and Tables

**Table 1:** Selected relevant studies of recent findings of EVs for the diagnosis and/or treatment of Alzheimer's disease.

Fluid of EVs	Origin cell-type of EVs	Isolation method	Purpose of the study	Target of study	Type of study	Main Results	Reference
Plasma	Neurons	ExoQuick^®^, isolation and immunoprecipitation (L1CAM)	Diagnosis	Synaptic proteins	Clinical	NDE levels of synapsynaptophysin, synaptopodin, synaptotagmin-2, and neurogranin were significantly lower in patients with FTD and AD. NDE levels of growth-associated protein 43 and synapsin 1 were reduced only in patients with AD.	Goetzl *et al.* 2016
Plasma	Neurons	ExoQuick,^®^ isolation and immunoprecipitation (L1CAM)	Diagnosis	NPTX2, NRXN2α, AMPA4 and NLGN1	Clinical	The NDE-derived proteins were significantly decreased in AD dementia. Diminished levels of AMPA4 and NLGN1 in NDEs correlated with the extent of cognitive loss. In a preclinical period, the NDE levels of all but NPTX2 were significantly lower than those of matched controls, and levels of all proteins significantly declined with the dementia conversion.	Goetzl *et al.* 2018
Plasma	Total	-	Diagnosis	Neurogranin	Clinical	Ng levels increased in CSF, but decreased in blood plasma exosomes of patients with AD and MCI-AD, and were highly associated with cognitive decline.	Liu *et al.* 2020
Plasma	Total	Ultracentrifugation	Diagnosis	Neurology and Inflammation proteins	Clinical	Several proteins and their ratios could distinguish cognitively affected from healthy individuals. For plasma TGF-α | CCL20 (AUC = 0.96, 95% CI = 0.88-1.00, p = 0.001) and EVs CLEC1B | CCL11 (AUC = 0.95, 95% CI = 0.86-1.00, p = 0.001) showed diagnostic capabilities.	Nielsen et al. 2020
Plasma	Neurons	ExoQuick^®^, isolation and immunoprecipitation (L1CAM)	Diagnosis	Protein cargo in NDEs	Clinical / Pre-clinical *(In Vivo)*	**Pre-Clinical**: Mice injected with plasma NDEs from ADC patients displayed increased p-Tau positive cells in the CA1 region of the hippocampus. **Clinical:** Plasma NDE levels of P-T181-tau, P-S396-tau, and Aβ_1-42_ were significantly higher, whereas neurogranin and the repressor element 1-silencing transcription factor were significantly lower in AD and MCI converting to AD patients.	Winston *et al.* 2016
Plasma	Neurons	ExoQuick^®^, isolation and immunoprecipitation (L1CAM)	Treatment	GHRH	Clinical	Exo-derived Aβ_1-42_, neurogranin, synaptophysin, synaptotagmin, and synaptopodin showed the highest diagnostic accuracy for distinguishing between CNC and MCI patients.	Winston *et al.* 2018
Plasma	Astrocytes	ExoQuick^®^, isolation and immunoprecipitation (GLAST/ACSA-1)	Diagnosis	C1q, C4b, factor D, fragment Bb, C5b, C3b, C5b-C9,CD46, CD59, and type 1 complement receptor	Clinical	ADE levels of complement proteins were significantly higher in patients with MCIc than those with MCIs. ADE levels of inhibitory CPs decay-accelerating were significantly lower in patients with MCIc than those with MCIc.	Winston *et al.* 2019
Plasma	Neurons	ExoQuick^®^, isolation and immunoprecipitation (L1CAM)	Prognosis	pS312-IRS-1 and pY-IRS-1	Clinical	The change from baseline in insulin signaling molecules in neuronal-enriched EVs in response to an experimental treatment (20 IU of intranasal insulin) is associated with a change in cognition, which suggests engagement of the insulin cascade in the brain.	Mustapic *et al.* 2019
Serum	Neurons	Total Exosome Isolation kit (Invitrogen Life Technologies)	Diagnosis	miRNA-135a, -193b and 384	Clinical	Both serum exosome miR-135a and miR-384 were up-regulated while miR-193b was down-regulated in serum of AD patients compared with that of normal controls. Exosome miR-384 was the best among the three miRNAs to discriminate AD, VaD, and PDD. Combination of miR-135a, -193b, and -384 was proved to be better than a particular one for early AD diagnosis	Yang *et al.* 2018
Serum, CSF and culture medium	Total	Total exosome Isolation kit (Invitrogen Life Technologies)	Diagnosis	miRNA-193b in ABCA1-labeled exosomes	Preclinical ( *In vivo* and *in vitro*)/ Clinical	ABCA1 and ABCA1-Labeled Exosomes Are Detected at High Levels in Neurons. ABCA1-Labeled Exosomal miR-193b Is Abundant in Serum and CSF from AD Model Mice. ABCA1-Labeled Exosomal miR-193b Levels Are Elevated in the CSF and Serum of AD Patients.	Liu *et al.* 2021
Saliva	Total	Ultracentrifugation	Diagnosis	Salivary exosomes	Clinical	Significant differences in salivary exosomes concentration among the groups of cognitively impaired and AD patients compared to the healthy control cohort.	Rani *et al.* 2021

**Ab1-42**, beta amiloide peptide; **ABCA1,** ATP Binding Cassette Subfamily A Member 1; **AD**, Alzheimer's disease;** ADC**, converting to AD; **ADEs**, astrocyt-derived exosome; **AMPA4**, GluA4-containing glutamate receptor; **CA1,** cornu ammonis 1**; CPs**, Levels of complement proteins; **CNC**; cognitively normal controls; **CSF**, cerebrospinal fluid**; C1q, C4b,** Complement Protein of classical pathway; **C5b, C3b, and C5b-C9,** Complement Protein of both patways; **CD46,CD59, and type 1 complement receptor,** Proteins of the inhibitory complement pathway;** CDAT**; dementia of the Alzheimer type; **factor D and fragment Bb,** Complement Protein of the alternative pathway;** FTD,** frontotemporal dementia**; GHRH**, Growth hormone-releasing hormone; **MCI**, mild cognitive impairment; **MCIC**, MCI converting to dementia within 3 years; **MCIS**, MCI remaining stable over 3 years; **NDE**, neuronal-derived exosomes; **NG**, neurogranin; **NLGN**1, neuroligin 1; **NPTX2,** neuronal pentraxin 2; **NRXN2α**, neurexin 2α; **PDD**, Parkinson's disease with dementia; **pS312-IRS-**1, Insulin Receptor Substrate 1 Phospho-Ser312; **P-T181-tau**, phospho tau T181; **P-S396-tau**, phospho tau S396;** pY-IRS-1**, Insulin Receptor Substrate; **SCD**; Subjective cognitive decline; **VaD**, vascular dementia;

**Table 2:** Selected relevant studies of recent findings of EVs for the diagnosis and/or treatment of Parkinson's Disease.

Fluid of EVs	Origin cell-type of EVs	Isolation method	Purpose of the study	Target of study	Type of study	Main Results	Reference
Plasma	Neurons	Centrifugation and immunoprecipitation (L1CAM)	Diagnosis	α-syn	Clinical / Pre-clinical *(In Vivo)*	**Pre-Clinical:** CSF α-syn was readily transported to blood, with a small portion being contained in exosomes. **Clinical:** The study found that in contrast to CSF α-syn concentrations, the levels of plasma exosomal α-syn were substantially higher in PD patients, suggesting an increased efflux of the protein to the peripheral blood of these patients	Shi *et al.* 2014
Plasma	Total	1) Total exosome Isolation kit (Invitrogen Life Technologies) 2) Differential centrifugation	Diagnosis	α-syn and p-α-syn	Clinical	α-syn and p-α-syn in the plasma exosomes of PD patients showed poor solubility after Protease K treatment.	Zheng *et al.* 2021
Plasma	Neurons	Centrifugation and immunoprecipitation (L1CAM)	Monitoring	NDEs	Clinical	Exenatide-related changes in EV biomarkers were significantly associated with clinical improvements.	Athauda *et al.* 2019
Plasma	Total	ExoQuick^®^-TC kit	Treatment	miRNA-137 and OXR1	Pre-clinical *(In vitro* and *In Vivo)*	Down-regulation of exosomal miR-137 alleviates oxidative stress injury in PD by up-regulating OXR1.	Jianj *et al.* 2019
Culture medium	ASCs	Ultracentrifugation and ExoQuick^®^-TC reagent	Treatment	miRNA-188-3p	Pre-clinical *(In Vivo* and *In Vitro)*	miRNA-188-3p-enriched exosome treatment suppressed autophagy and pyroptosis, whereas increased proliferation via targeting CDK5 and NLRP3 in PD mice and MN9D cells were observed.	Li *et al.* 2019
Culture medium	BMSCs	Ultracentrifugation	Treatment	EXO-ASO4	Pre-clinical *(In Vivo* and *In Vitro)*	***In Vitro:*** Exo-ASO4 also significantly attenuated α-syn aggregation induced by pre-formed α-syn fibrils. ***In Vivo:*** Exo-ASO4 intracerebroventricular injection into the brains of α-syn A53T mice significantly decreased the expression of α-syn and attenuated its aggregation. Furthermore, it ameliorated the degeneration of dopaminergic neurons in these mice and showed significantly improved locomotor functions.	Yang *et al.* 2021

**ASCs**, Adipose-derived mesenchymal stem cells;** ASO(s)**, Antisense oligonucleotide(s); **BMSCs,** Bone marrow Mesechymal stem cells; **CDK5**, cell division protein kinase 5; **CSF,** cerebrospinal fluid; **DA**, dopaminergic; EV; extracellular vesicles; **Exo**, Exosomes; **L1**, L1 cell adhesion molecule; **NLRP3**, Nucleotide-binding and oligomerization domain-like receptor family pyrin domain containing 3; **PD**, Parkinson's disease; **ROS**, reactive oxygen species; **SOD**, superoxide dismutase; **OXR1**, oxidation resistance 1; **α-syn**, α-sinuclein;** p-α-syn**, Ser129 phosphorylated α-sinuclein.

**Table 3:** Selected relevant studies of recent findings of EVs for the diagnosis and/or treatment of Multiple Sclerosis.

Fluid of EVs	Origin cell-type of EVs	Isolation method	Purpose of the study	Target of study	Type of study	Main Results	Reference
Serum	Total	Size exclusion chromatography + qEV size exclusion columns	Diagnosis & monitoring	miRNAs	Clinical	A group of nine miRNAs (miR-15b-5p, miR-23a-3p, miR-223-3p, miR-374a-5p, miR-30b-5p, miR-433-3p, miR-485-3p, miR-342-3p, miR-432-5p) distinguished relapsing-remitting from progressive disease.	Ebrahimkhani et al. 2017
Serum	Total	Total exosome Isolation kit (Invitrogen Life Technologies)	Diagnosis	Macrophages	Clinical / Pre-clinical (*In vitro*)	Serum-derived exosomes of patients with MS expressed higher levels of EBV proteins than HC. Incubation with EBV(+) exosomes promoted MDMs activation and pro-inflammatory cytokines secretion.	Mrad et al. 2021
Culture medium	BMSCs	Ultracentrifugation	Treatment	Neuroinflammation	Pre-clinical *(In Vivo)*	Exosomes from BMSCs significantly decreased neural behavioural scores, neuroinflammation, and demyelination. In addition, exosomes increased the levels of IL-10 and TGF-β, whereas TNF-α and IL-12 levels decreased significantly.	Li *et al.* 2019
Culture medium	AMSCs	Exocib^®^ exosome isolation kit	Treatment	Immunomodulation	Pre-clinical *(In Vivo)*	Intranasal administration of MSC-SEV to EAE mice was more effective than the administration of MSC alone to reduce clinical scores and histological lesions of the CNS tissue.	Fathollari *et al.* 2021
Culture medium	MSCs	ExoQuick^®^-TC kit	Treatment	Myelin	Pre-clinical *(In Vivo* and I*n Vitro)*	*In Vitro:* The aptamer-exosome promoted the proliferation of OLN93 cell line. *In vivo:* The aptamer-exosome produced a robust suppression of inflammatory response as well as lowered demyelination lesion region in CNS, resulting in reduced severity of the disease in a C57BL/6 mice model.	Hosseini Shamili *et al.* 2019
Culture medium	Dendritic cells	ExoQuick^®^ Kit	Treatment	INF-γ	Pre-clinical *(In vivo)*	Nasally administered IFNγ-DC-Exos increased CNS myelination in vivo.	Pusic *et al.* 2014

**AMSCs**, Abdominal adipose tissue MSC; **BMSCs,** Bone marrow Mesechymal stem cells; **CNS**; central nervous system; **EAE**; Experimental autoimmune encephalomyelitis; **EBV**, Epstein-Barr virus; **INF-γ**, Interferon gamma;** IFNγ-DC-Exos**, low-level interferon gamma released exosomes; **MDMs**, monocyte-derived macrophages;** MSCs**, Mesechymal stem cells; **SEV**, Small extracellular vesicle.

**Table 4:** Selected relevant studies of recent findings of EVs for the diagnosis and/or treatment of Amyotrophic Lateral Sclerosis.

Fluid of EVs	Origin cell-type of EVs	Isolation method	Purpose of the study	Target of study	Type of study	Main Results	Reference
Culture medium	Astrocytes	Ultracentrifugation	Diagnose and treatment	SOD1	Pre-clinical *(In Vitro)*	Mutant SOD1 astrocytes release mutant SOD1-containing exosomes that are toxic for motor neurons. Results suggest that astrocyte-derived exosomes may have a role in disease spreading and motor neuron pathology.	Basso *et al.* 2013
Brain and spinal cord tissue	Astrocytes, neurons and microglia	Ultracentrifugation	Diagnose and treatment	SOD1	Pre-clinical *(In vivo)* and Clinical	Brain astrocytes and neurons, but not microglia, are the main EV source and are involved in the disease development thought their pathogenic disease-causing proteins.	Silverman et al. 2019
Culture medium	ASCs	PureExo^®^ Exosome isolation kit	Treatment	Oxidative stress	Pre-clinical *(In vitro)*	Exosomes were able to protect NSC-34 cells from oxidative damage and increase the cell viability.	Bonafede *et al.* 2016
Culture medium	ASCs	PureExo^®^ Exosome isolation kit	Treatment	Motor performance	Pre-clinical *(In vivo)*	ASC-derived exosomes targeted lesioned ALS regions, protected muscle, lumbar motoneurons and the neuromuscular junction, improved the motor performance, and decreased the glial cells activation.	Bonafede et el. 2020
Plasma	Total	EXOBead/EXOBuffer^®^	Diagnosis	NfL, pNfH and TDP-43	Clinical	Exosomal TDP-43 ratio was increasing along with follow-up.	Chen *et al.* 2020
CSF	Total	Size Exclusion Chromatography on Drip Column	Diagnosis	CSF proteins	Clinical	Three proteins were increased and 11 proteins were decreased in ALS patients in CSF exosomes. The protein with the greatest increase was INHAT repressor, which is closely associated with nucleolar function.	Hayashi *et al.* 2020
Plasma	Total	Differential centrifugation and filtration protocol	Diagnosis	Small EVs, large EVs and plasma	Clinical	Raman spectra showed that sporadic ALS patients have a different EVs lipid/protein content and less intense bands than healthy controls.	Morasso *et al.* 2020
Plasma	Total	Vn96 peptide isolation and centrifugation	Diagnosis	miRNA signature in extracellular vesicles	Clinical	Elevated levels of 5 miRNAs and reduced levels of 22 miRNAs in EVs. miRNAs with relevance to ALS were found to be deregulated: - Diagnosis: miR-9-5p, miR-183-5p, miR-338-3p, - Disability progresion:.miR-1246. MiR-15a-5p and miR-193a-5p	Saucier *et al.* 2019

**ALS**, amyotrophic lateral sclerosis; **ASCs,** adipose-derived stromal cells; **CSF**, Cerebrospinal fluid; **EVs**, extracellular vesicles; **lEVs,** large extracellular vesicles; **NIR**, INHAT repressor; **NFL**, Neurofilament light chain; **PALS**, persons diagnosed and living with ALS; **pNfH**, phosphorylated neurofilament heavy chain; **SOD1**, Copper-Zinc Superoxide Dismutase; **TDP-43**, exosomal TAR DNA-binding protein-43.

**Table 5:** Selected relevant studies of recent findings of EVs for the diagnosis and/or treatment of brain tumors.

Fluid of EVs	Origin cell-type of EVs	Isolation method	Purpose of the study	Target of study	Type of study	Main Results	Reference
Culture medium	GBM	Ultracentrifugation	Diagnosis and treatment	Glioma-derived exosomes	Pre-clinical *(In Vitro* and *In Vivo)*	Hypoxic glioma-derived exosomes (H-GDEs) markedly facilitated autophagy and M2-like macrophage polarization compared with normoxic glioma-derived exosomes (N-GDEs), which subsequently promoted glioma proliferation and migration *in vitro* and *in vivo*. In addition, interleukin 6 (IL-6) and miR-155-3p were highly expressed in H-GDEs.	Xu et al. 2021
Culture medium	GBM	Ultracentrifugation and filtration	Diagnosis and treatment	GDEs ad peripheral immune effectors	Pre-clinical *(In Vitro)*	The results suggest that malignant glioma-derived exosomes are restricted in their capacity to directly prime peripheral immunosuppression.	Iorgulescu *et al.* 2016
Culture medium	GBM	ExoQuick^®^TC TM	Treatment	VEGF	Pre-clinical *(In Vitro* and *In Vivo)*	VEGF-C isoform in GDEs had a role in GBM angiogenesis, stimulation of endothelial cell viability, migration, and tubulation.	Wang *et al.* 2021
Plasma	GBM	ExoQuick^®^ one-step precipitation solution	Diagnosis/ Treatment	miRNA-148a	Clinical / Pre-clincal *(In Vitro)*	miR-148a delivered by exosomes may promote cancer cell proliferation and metastasis via targeting CADM1 to activate STAT3 pathway. This highlight the predictor and therapeutic target role of exosomal miR- 148a for GBM patients.	Cai *et al.* 2020
Serum	GBM	ExoQuick^®^ precipitation	Diagnosis & monitoring	miRNA signature	Clinical	Exosomes were isolated before and after radiotherapy and exosomes-derived miRNAs sequencing was performed. A total of 18 up-regulated differentially expressed miRNAs and 16 down-regulated differentially expressed miRNAs were identified. These miRNAs were shown to play a crucial role in glioma by regulating targets and affect the occurrence and development of the disease.	Li *et al.* 2020
Serum / Culture medium	GBM	ExoQuick^®^ exosome precipitation kit	Prognosis	Exosomal CircNFIX on TMZ chemoresistance	Clinical / Pre-clinical *(In Vivo* and *In Vitro)*	Pre-clinical : *In Vitro:* Exosomal CircNFIX confers TMZ resistance in glioma cells *In Vivo:* Exosomal CircNFIX enhances tumor growth and TMZ resistance in vivo **Clinical:** **-** Serum exosomal CircNFIX expression is up-regulated in TMZ-resistant patients	Ding *et al.* 2020
Bone marrow	Neutrophils from bone marrow	Ultracentrifugation	Treatment	Exosomes as drug carriers	Pre-clinical (*In vivo*)	Exosomes were isolated from neutrophils extracts of mice bone marrow. NEs-Exos penetrated the BBB and migrated into the brain. NEs-Exos responded chemotactically to inflammatory stimuli and targeted infiltrating tumor cells. DOX-NEs-Exos suppressed tumor growth and prolong survival time in the glioma *in vivo* models.	Wang *et al.* 2021

**BBB**, blood brain barrier; **CADM1**, Cell adhesion molecule 1; **CDK6**, cyclin dependent kinase 6; **CircNFIX**, circRNA nuclear factor I X; **DE**, differentially expressed; **DOX**, doxorubicin; **GBM**, glioblastoma; **GDEs**, glioma-derived exosomes: **INF-PA**, invasive NF-PA; **NEs-Exos**, neutrophil-exosomes; **NF-PAs**, invasiveness of non-functioning pituitary adenomas;** NNF-PA**, noninvasive NF-PA; **RHOU**, ras homolog family member U; **SPIRE2**, spire type actin nucleation factor 2; **STAT3**,Signal transducer and activator of transcription 3;**TMZ,** temozolomide;**VEGF,** vascular endothelial growth factor

## Abbreviations

AD: Alzheimer's disease
ADEs: astrocyte-derived exosomes
ALS: amyotrophic lateral sclerosis
EAE: experimental autoimmune encephalomyelitis
EVs: extracellular vesicles
GBM: Glioblastoma
MDEs: microglia-derived exosomes
MS: multiple sclerosis
MVBs: multivesicular bodies
NDEs: neuronal-derived exosomes
PD: Parkinson's disease
VD: vascular dementia
